# Thermal insulation property of graphene/polymer coated textile based multi-layer fabric heating element with aramid fabric

**DOI:** 10.1038/s41598-020-74339-8

**Published:** 2020-10-16

**Authors:** Hyelim Kim, Han Seong Kim, Sunhee Lee

**Affiliations:** 1 Research Institute of Convergence Design, Dong-A University, Busan, 49315 Republic of Korea; 2 Department of Organic Material Science and Engineering, Pusan National University, Busan, 46241 Republic of Korea; 3 Department of Fashion Design, Dong-A University, Busan, 49315 Republic of Korea

**Keywords:** Materials science, Nanoscience and technology

## Abstract

This study investigated the thermal insulation properties based on electrical heating test of graphene-based multi-layer fabric heating elements to confirm the possibility of application for fabric heating element for protective clothing. Four layers were designed as layers of outer, filler, electrical heating textile, and lining. The outer fabrics used two different densities of aramid woven fabrics (LD_ARW and HD_ARW), an aramid knit (AR_KT), and nonwoven (AR_NW). Fabricated graphene/polymer coated electrical heating textile (GR) exhibits a surface temperature of about 85 °C, a current of 0.12 A, and a power of 3 W when 30 V is applied. As composed with 4-layer, the surface temperature of LD_ARW and HD_ARW used as the outer for sample indicated less than 50 °C, due to their excellent heat resistance property; whereas, when AR_KT and AR_NW were used, the temperature was about 50 °C. This is because their fine fibers form high porosity that can entrap air. As a result of the thermal insulation properties, the temperature difference of each layer was in the order ΔT_(GR-N3)_ < ΔT_(GR-Lining)_ < ΔT_(GR-Outer)_. In particular, when AR_NW was used as the outer fabric, ΔT_(GR-Outer)_ was decreased by about 10 °C, compared with that of the other outer fabric. By the effect of relative humidity under dry 25% RH and comfortable 55% RH, the temperature difference was decreased under 55% RH; thus, the thermal insulation property was improved under comfortable humidity condition. Therefore, the best thermal insulation performance was exhibited when AR_NW was used as outer under 55% RH, and it is expected to expand its application to fabric heating element for protective clothing.

## Introduction

A cold environment results in a decrease in body temperature, and prolonged exposure causes cold-related illness, leading to numbness, frostbite, hypothermia, and eventually death^1–3^. To maintain and protect the body temperature of the human body exposed to cold environments, clothing plays the role of a passive insulation layer, and is used to protect the human body from severe cold. In general, clothes have excellent thermal insulation from a stationary air layer that is formed between the fabric layers. In particular for winter clothing, thermal insulation properties play a crucial role for human heat maintenance. Thus, winter clothing consists of three or more layers of multilayer structure with different functions, in order to ensure thermal comfort in winter outdoor climatic conditions: (1) Outer layer: Protection against external environment, (2) middle thermo-insulating layer: thermal-insulation nonwoven or other thermal insulating material that protects the human body from excessive heat loss, (3) inner layer-lining: layer nearest to the human body that exchanges moisture and air between the human body and the environment^4^. Passive and active methods are used to reduce heat loss through clothes. A passive method to increase thermal insulation is to increase the air layers of clothing using down filler or multi-layer clothes^5^. In addition, when multilayer clothing is configured, the amount of air content varies, depending on the fabric type, structure, thickness, density, weight, and porosity of each layer, thereby affecting the thermal insulation property. In particular, in cold protective clothing for winter, a very important role is played by the middle layer, the basic function of which is to protect the human body against chilling. Different kinds of textile materials are used as the middle thermal insulating layer of multilayer clothing^6–10^. An active method can be defined by providing extra heat using heating textiles or units, such as far-infrared, solar, chemically heated textiles, phase change material textiles or electrically-aided heating units^11–13^.

Electrical heating textile is one of the active methods for increasing thermal insulation value in cold protective clothing. It is mainly manufactured by conductive materials, such as copper, silver, or carbon-nano materials, on yarn or untreated fabrics^14^. Generally, electrical heating garments mainly used by copper and stainless-steel materials. These materials are electrically stable due to the electrical resistance does not change during stretching, but those are easy to break as brittle properties. Accordingly, for providing stretchable to long metal wire, electrical heating textiles are manufactured by bending as a wound type, or coating nanoparticles on yarns. In the case of manufacturing a wound type with metal wire, there is a disadvantage that excessive heat is generated in the bending portion and can cause damage, and in the case of metal nanoparticles which form a conducive network are eliminated, thereby deteriorating performance. In recent, textile heating systems by coating with conductive inks or pastes such as carbon nano-based materials have a huge advantage due to their ability to bend and flex, and hence could provide heating effects for irregular geometries, such as tubular and spherical structures^15^. In addition, since mass production is possible, they can be applied to various fields. Recently, the electrical heating textile is applied to heating garments, outdoor blankets, and sleeping bags, and research on electric heating performance, thermal insulation properties, and wearability has been continuously conducted^16–22^. Shin et al.^16^ have reported the evaluation of the effects of heating protocols using graphene-heated clothing in cold environment. The graphene heater was located in the pocket of the upper back or chest of underwear. It was reported that graphene heater at upper back heating could increase the temperature and maintain the body temperature better, compared with chest heating. Zhang et al.^18^ have prepared a smart electrically heated sleeping bag to improve wearer’s thermal foot comfort in a cold ambient environment. The smart sleeping bag was aimed to maintain human feet within the thermoneutral range (25.0–34.0) °C. A smart electrically-heated sleeping bag consisted of nylon shell, nylon lining, filled with polyester fiber and flexible carbon polymer heating pad. Finally, it was developed to be able to provide wearers with an 8 h comfortable sleep in a cold environment. Lee and Jeong^22^ have evaluated the heating performance of commercial heated vests of three types. The thermal images and the temperature between body and heating vests was investigated by IR camera. The results of evaluating the heating performance of three types of commercial heating vests differing in the type of material and the heating element constituting the heating garment were as follows. The film-type heating element showed the best performance when works itself. On the other hand, the human body wearing test results in the cold environment showed that the heat protection effect of the commercial heating vest with the filler and nonwoven-type heating element applied to the vest was the best. To design an excellent heating garment, it was reported that both the heating performance of the heating element and the thermal insulation properties of the heating garment itself should be considered.

Therefore, in this present study, in order to apply the horseshoe pattern electrical heating textile coated with graphene/polymer manufactured in our previous study^24^ as protective garment, a graphene-based multi-layer fabric heating element was designed based on aramid-based fabric. After that, the surface temperature of each layer and the thermal insulation performance inside the layer of the designed samples were analyzed based on the electric heating test under 55% RH, which is the comfortable humidity condition. Moreover, in consideration of the environmental conditions used in extreme condition in winter, the thermal insulation performance based on electrical heating test was also measured under dry condition of 25% RH. The specific goals were as follows. First, the graphene-based multi-layer fabric heating element was finally intended to consist of four layers. The fabrics used in each layer were: (1) Outer fabric: two types of waterproof fabrics and two types of aramid woven fabrics, aramid knit and aramid nonwoven fabric. (2) Filler: neoprene textile (3) horseshoe pattern electrical heating textile coated by graphene/polymer (below GR). (4) Lining: waterproof brushed PET fabric. After that, each sample was composed of 2-layer, 3-layer, and 4-layer textile based on GR as 1-layer; and then the temperature change and difference of each layer was examined by electrical heating test. Finally, to investigate the thermal insulation properties from the effect of relative humidity, four types of graphene-based multi-layer fabric heating element with aramid-based outer fabrics were investigated based on the electrical heating test at the conditions of (25 and 55) % RH.

## Results and discussion

**Figure 1:**
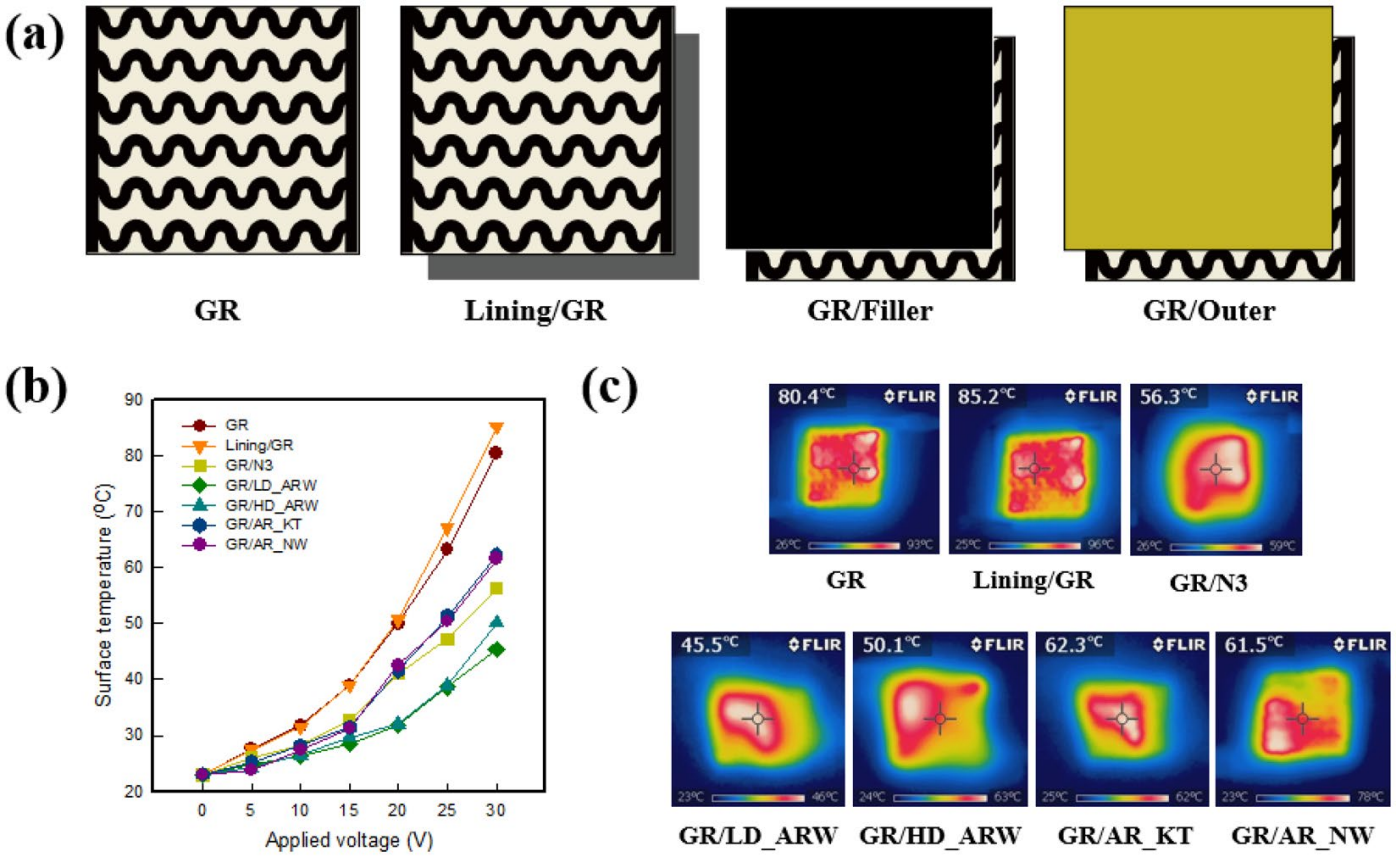
(**a**) Measuring layer, (**b**) Surface temperature of single layer and double layer of graphene-coated fabric-based fabric heating element with various applied voltages, and (**c**) IR images at 30 V.

Figure 1 represent the surface temperature of graphene-based multi-layer fabric heating elements with various fabric types and layers by thermal image camera. Figure 1a illustrates the graphene/polymer coated cotton fabric (below GR) and double-layer for each fabric for measuring surface temperature. Figure 1b shows the surface temperature of each sample increased as the applied voltage increased from (5 to 30) V, according to Joule’s law^23^. When (20 and 30) V was applied to the GR, which was used as a direct heating supply, it showed about (50.2 and 80.4) °C, respectively, and the performance of the fabric heating element was confirmed. GR sample which is an active heating supply is coated with a graphene/polymer composite on fabric. When voltage is applied to GR, the electrical heating textiles which composed of carbon nanomaterial the free electrons are randomly disordered and without direction as the current flows, and they appear as a resistance heating when they collide. According to Joule's law, when the voltage (*V*) increases, the current (*I*) increases proportionally, thus, the power (*P*) also increases^24^. The surface temperature after layering the brushed waterproof polyester fabric (below WPF_B) under GR by lining tended to be about (4.0–5.0) °C higher than when GR was used as single layer after 25 V, and the surface temperature of the Lining/GR was about 85.2 °C when 30 V was applied, as shown in the IR image (Fig. 1c). Generally, the brush finishing was reported to enhance the resistance of the textile material to atmospheric agents by improving thermal insulation and warmth, which was provided by the insulating air cells in the nap^25^. For this reason, the air layers warmed by GR could be continuously heat diffused in the fabric, because the nap in the WPF_B forms fine porosity. Thus, it is confirmed that an air layer capable of heat diffusion can be generated, compared with when GR is used as a single layer. On the other hand, when layering the N3, which is filler, and outer fabrics for LD_ARW, HD_ARW, AR_KT, or AR_NW on top of GR, they showed the opposite tendency to the lining. After applying 30 V to GR, when layered with N3, it was about 56.3 °C. Also, when layering two aramid woven fabrics, LD_ARW and HD_ARW, it was found to be about (45.5 and 50.1) °C; and when AR_KT and AR_NW were used, they showed surface temperatures of about (62.3 and 61.5) °C, respectively. This result shows that the use of AR_KT, and AR_NW on the GR decreased about 20.0 °C compared with when only GR was used, and decreased about (25.0–30.0) °C when using N3, LD_ARW, and HD_ARW. This was found to be related to the density, thickness, and fabric structure that make up the fabric^4,5^. When AR_KT and AR_NW are layered on the GR, it is confirmed that both fabrics can maintain a warm air layer generated by GR, due to possessing many regions that contain air layers in those materials, and thus it shows higher surface temperature than the other outer fabrics. N3 used as a filler has a thickness that is about three times thicker than the other fabrics, while it can contain many air layers due to the pores in neoprene, and the temperature of about 56.3 °C was found, due to the possible heat diffusion air layer. LD_ARW and HD_ARW showed lower surface temperature than other fabrics, which confirmed that the temperature was low, due to the strong heat resistance of the aramid fabric.

**Figure 2:**
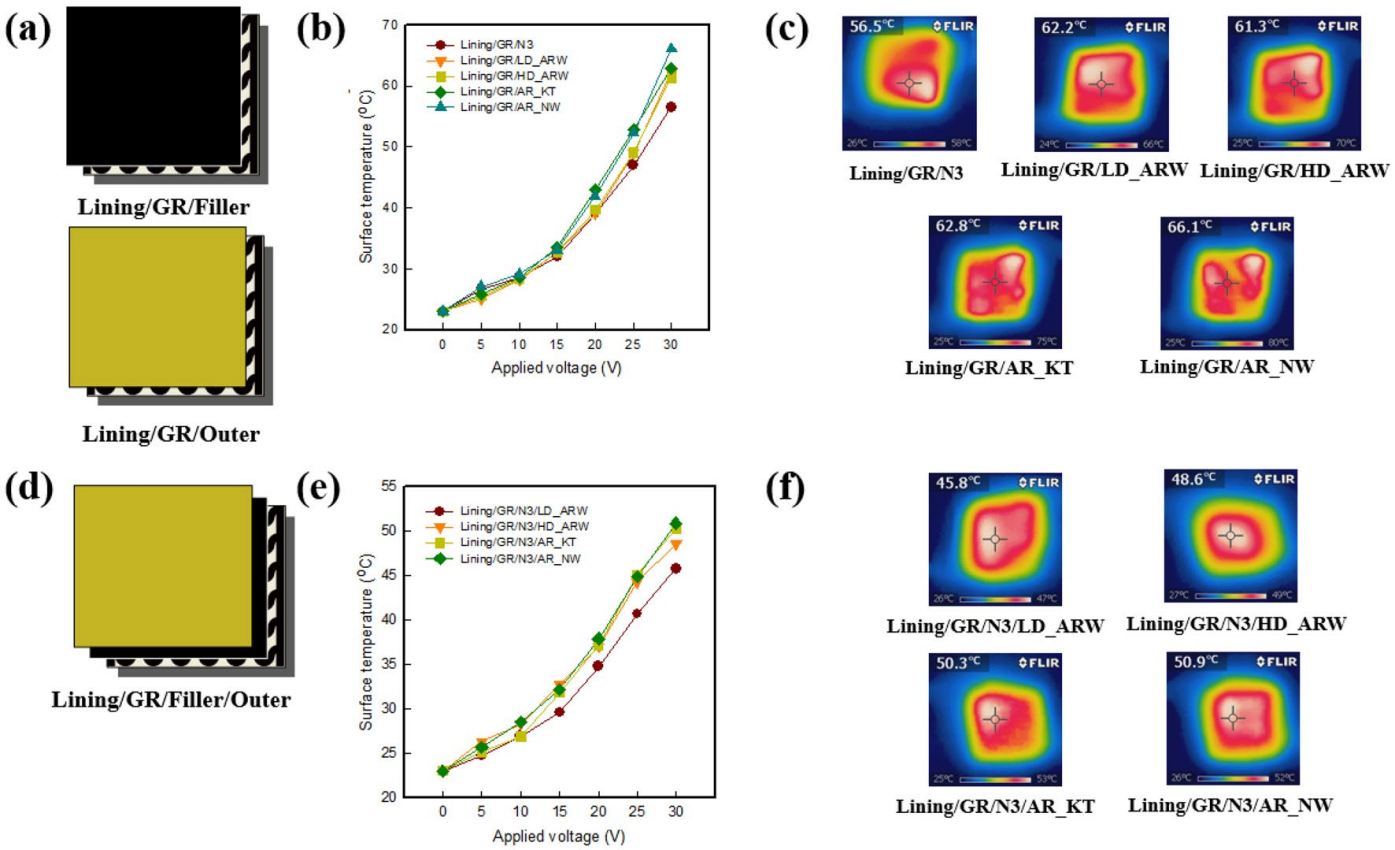
(**a**), (**d**) Measuring layer of triple- and quadruple-layer samples, (**b**), (**e**) Surface temperature of triple- and quadruple-layers of samples with various applied voltages and (**c**), (**f**) IR images of each sample at 30 V.

Figure 2 shows the surface temperature and IR images of the graphene-based triple- and quadruple-layer fabric heating elements layered on the lining/GR and lining/GR/filler based with various fabric types. Samples were designed as shown in Fig. 2a,d. Figure 2b shows that the surface temperature of the triple-layer samples after layering with lining was about 60.0 °C. It was confirmed that the electrical heating performance was improved, compared to the samples that were not layered. When 30 V was applied to the samples, the surface temperature of Lining/GR/N3 was 56.5 °C, and the surface temperature of Lining/GR with two aramid woven fabrics of LD_ARW and HD_ARW were about (62.2 and 61.3) °C, while the surface temperature of both Lining/GR/AR_KT and Lining/GR/AR_NW was about (66.1 and 62.8) °C, respectively (Fig. 2c). It was confirmed that the nap of the fabric used for lining increased the area of porosity where the stationary air layer could be present, thus raising the temperature^25^. Also, the difference in the surface temperature was affected by some heat diffusion and heat transfer, depending on the fabric structure. The thicker the fabric, the lower the heat transfer, so it appears to have the lowest surface temperature with N3. In addition, when AR_KT and AR_NW are used to the sample, the temperature is increased, because the fabric structure may contain high porosity and air layer^4–6^. Thus, it is confirmed that the characteristics of the brushed fabric in lining may contain an air layer, which is responsible for the surface temperature rise as the warm air layer formed by the GR is entrapped.

Figure 2e shows the surface temperature of graphene-based quadruple-layer fabric heating. As shown in the previous results, the surface temperature also increased as the applied voltage was increased, whereas the surface temperature of the graphene-based quadruple-layer fabric heating element was decreased by about 10.0 °C compared with the triple-layer samples, thus the temperature range appeared to be between (45.0 and 51.0) °C. When 30 V was applied, the surface temperature of Lining/GR/N3/LD_ARW and Lining/GR/N3/HD_ARW using two aramid woven fabrics was about (45.8 and 48.6) °C. As the HD_ARW was used as outer layer, the surface temperature was raised by about 3.0 °C, compared with LD_ARW. These results appear to be due to the increase in fabric density, which increases the amount of air entrapped in the fabric as the fabric tightens; thus it was found that the heat loss was reduced by decreasing the air circulation in the fabric^6^. Lining/GR/N3/AR_KT and Lining/GR/N3/AR_NW showed the higher surface temperatures of about (50.3 and 50.9) °C, respectively, compared with when the two aramid fabrics were used. This is because knits generally have a bulkier property than woven fabrics, and thus could contain more air layers in the fabric, and the nonwoven could also accumulate more layers of air in porous structures made of the fine fibers that exist in bulky fabric structures. Therefore, the graphene-based multi-layer fabric heating element was improved by adding lining when layering, and the surface temperature was different according to the fabric structure and factors of the outer fabric.

**Figure 3:**
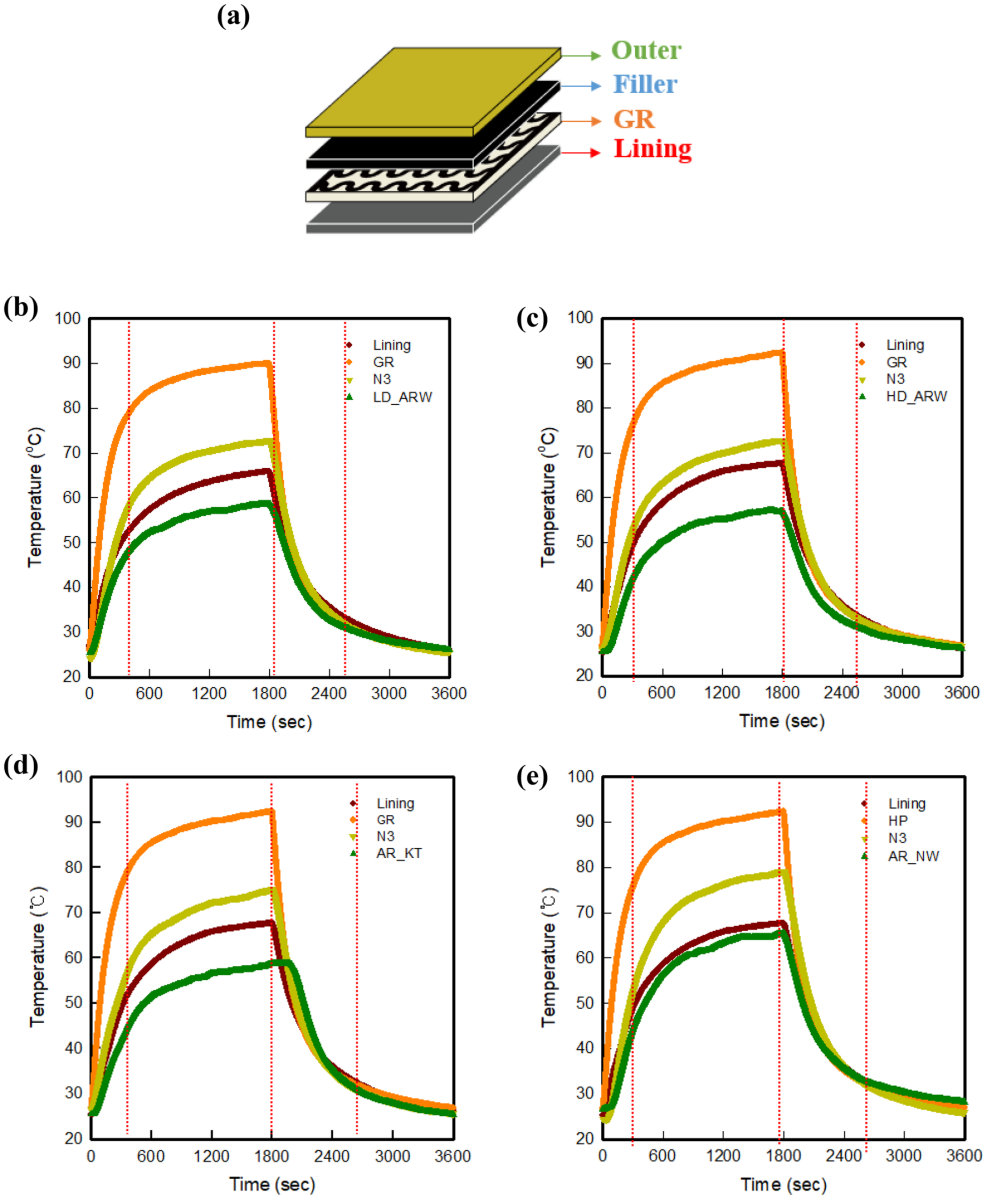
(**a**) Measured layer and time dependent on surface temperature of each layer for graphene-based multi-layer fabric heating element with various outer fabrics, (**b**) LD_ARW, (**c**) HD_ARW, (**d**) AR_KT, and (**e**) AR_NW.

Figure 3 shows the temperature variation of each layer according to the fabric type of the final quadruple-layer graphene-based multi-layer fabric heating element. Based on Lining/GR/N3, the four different aramid fabrics were used as outer materials. At that time, the electrical heating temperature and the electrical heating behavior of each layer were measured by temperature sensor depending on the time (Fig. 3a). For measurement, the applied voltage was fixed at 30 V, because the final surface temperature of the graphene-based quadruple-layer fabric heating element indicated about 50.0 °C, which is a comfortable temperature of the human body. The electrical heating behavior of samples was measured for 30 min, and recorded until the equilibrium state, after turning off the power.

As shown in Fig. 3a–e, the surface temperature of each graphene-based multi-layer fabric heating element composed of quadruple-layer was found to be in the order Outer < Lining < Filler < Electrical heating textile. After applying the voltage, the temperature of the initial stage increased rapidly in each layer, and the plateau behavior was indicated until 30 min, before turning off the power. Among the quadruple-layers, GR was a direct source of heating. The temperature of GR rapidly rose at the initial stage more than the lining, filler, and outer fabric, and reached about 85.0 °C within about 3 min, as seen in the IR images. After that, the temperature of lining fabric, a layer located close to GR, rose rapidly, and plateau behavior was observed from (5 to 30) min. In the case of the filler, although it was the upper layer that was in direct contact with the GR, the initial temperature rise was slower than that of the lining. In addition, the time to reach the plateau region appeared after 10 min, thus it was confirmed that this region started later than for the other fabrics. The outer layer showed electrical heating behavior that was similar to that of the filler, while the temperature difference was about 20.0 °C. It was found that the surface temperature decreases because the outer material located in the outermost layer, and thus heat is not directly transmitted from the GR, which is the heating supply.

In addition, the variation of temperature between layers depending on the type of outer fabric is also indicated through Fig. 3b–e. As shown in Fig. 3b,c, the temperature tendency of GR did not show a significant change after 10 min, but the lining and N3 temperature slightly increased. It was confirmed that the aramid-based fabric prevents heat-resistance by dissipating the heat to the outside, but due to the layer underneath, the aramid-based fabric can retain the air layer. Thus, the air temperature warms up, and the surface temperature tends to increase slightly over time. Among the samples, in the case of using AR_NW as the outer, it was confirmed that the temperature of the N3 and outer layer was increased by about 10.0 °C, compared with when using the other fabrics. It was found that when AR_NW is used as the outer material, much of the porosity formed by fine fibers had risen in temperature while entrapped in warm air (Fig. 3d–e).

**Figure 4:**
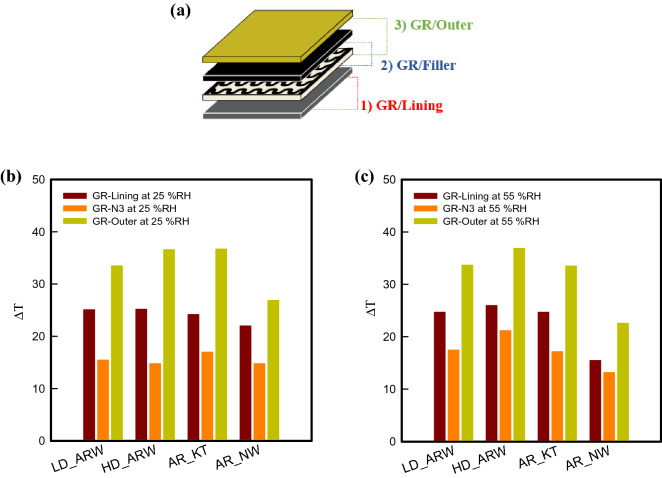
(**a**) Measured layer, and (**b**) the temperature difference between GR and each layer in a graphene-based multi-layer fabric heating element under 25% RH (left) and 55% RH (right).

Figure 4 represents the temperature difference between GR and each layer in a graphene-based multi-layer fabric heating element to analyze thermal insulation properties based on an electrical heating test under (25 and 55) % RH conditions at 20 °C. When analyzed by layer, the four types of graphene-based multi-layer fabric heating elements showed the smallest ΔT _(GR-N3)_ between GR, which is an electrical heating fabric, and N3, which is a filler. Since it is the fabric layer that is in direct contact with the GR, the heat transfer is easiest from GR to filler, and thus the temperature deviation is confirmed to be small. Next, the temperature difference between GR and lining, which is ΔT_(GR-Lining)_, and the temperature difference between GR and outer fabric, which is ΔT_(GR-Outer)_, tended to increase. It was found that the temperature difference increases due to increase of heat loss as the layer position and the fabric layer increase ^1^.

In addition, it could be seen that the temperature difference for each fabric layer shows different results, depending on the type of outer fabric and humidity. For graphene-based multi-layer fabric heating elements using LD_ARW as the outer layer fabric, ΔT_(GR-Lining)_, ΔT_(GR-N3)_, and ΔT_(GR-Outer)_ were (24.7, 17.5, and 33.7) °C, respectively; and for samples using HD_ARW as the outer layer fabric, were (26.0, 21.2, and 36.9) °C, respectively. The temperature difference with N3 tended to decrease than Lining or outer layer. It was shown that the temperature of N3, which is the closest layer to GR, decreases as warm air from GR, which generated the heat, was entrapped in N3 inside of the aramid woven fabrics. When AR_KT was used as the outer layer fabric, it showed a similar tendency, ΔT_(GR-Lining)_, ΔT_(GR-N3)_, and ΔT_(GR-Outer)_ were (24.7, 17.2, and 33.5) °C, respectively. On the other hand, when AR_NW was used as the outer layer fabric, it was confirmed that the temperature difference appeared the smallest between GR and each layer, and the values of ΔT_(GR-Lining)_, ΔT_(GR-N3)_, and ΔT_(GR-Outer)_ were (15.5, 13.2, and 22.6) °C, respectively. It was found that due to the high porosity of the fine fibers, which is a structural feature of nonwoven fabric, generated heat from the GR could be accumulated in N3. After that, it was confirmed that the accumulated heat was also kept in the N3, thus, the temperature difference of each layer decreases as the temperature rises. In addition, since the nonwoven fabric that has better heat diffusion and heat transfer capability than the woven fabric structure was used as the outer layer fabric, it was represented that the surface temperature easily increased, and the temperature difference decreased, compared with that of the other outer layer fabrics. Also, it was confirmed that the temperature difference increased as the surface temperature of LD_ARW and HD_ARW decreased due to using the aramid woven fabric, which possessed high thermal conductivity as the outer layer fabric. Therefore, the temperature difference between GR and each layer of the graphene-based multi-layer fabric heating element was different, according to the type of outer fabric. Also, when the fabric using AR_NW as the outer layer fabric was used, it was confirmed that the thermal insulation properties were excellent, due to the smallest difference of temperature.

Figure 4b represents the ΔT value of the graphene-based multi-layer fabric heating element under 25% RH. When LD_ARW was used as the outer layer under 25% RH, ΔT_(GR-Lining)_, ΔT_(GR-N3)_, and ΔT_(GR-Outer)_ were (25.1, 15.5, and 33.5) °C, respectively. Also, for the sample using HD_ARW as the outer fabric, ΔT_(GR-Lining)_, ΔT_(GR-N3)_, and ΔT_(GR-Outer)_ were (25.2, 14.8, and 36.6) °C, respectively. In addition, ΔT_(GR-Lining)_, ΔT_(GR-N3)_, and ΔT_(GR-Outer)_ of samples using AR_KT as the outer layer at 25% RH were (24.2, 17.0, and 36.7) °C, respectively. For samples using AR_NW as the outer layer, ΔT_(GR-Lining)_, ΔT_(GR-N3)_, and ΔT_(GR-Outer)_ were (24.0, 14.8, and 26.9) °C, respectively, at 25% RH.

As shown in Fig. 4c, for graphene-based multi-layer fabric heating elements using LD_ARW as the outer layer fabric, ΔT_(GR-Lining)_, ΔT_(GR-N3)_, and ΔT_(GR-Outer)_ under 55% RH condition were (24.7, 17.5, and 33.7) °C, respectively; and for samples using HD_ARW as the outer layer fabric, were (26.0, 21.2, and 36.9) °C, respectively. When compared by fabric types, it was confirmed that the temperature difference increased as the surface temperature of LD_ARW and HD_ARW decreased, due to the use of aramid woven fabric that possessed high thermal conductivity as the outer layer fabric. Also, when AR_KT was used as the outer layer fabric, it showed a similar tendency, and ΔT_(GR-Lining)_, ΔT_(GR-N3)_, and ΔT_(GR-Outer)_ at 55% RH condition were (24.7, 17.2, and 33.5) °C, respectively. On the other hand, when AR_NW was used as the outer layer fabric, it was confirmed that the temperature difference appeared the smallest between GR and each layer, and the value of ΔT_(GR-Lining)_, ΔT_(GR-N3)_, and ΔT_(GR-Outer)_ under 55% RH condition were (15.5, 13.2, and 22.6) °C, respectively. It was found that due to the high porosity caused by fine fibers, which is a structural feature of nonwoven fabric, generated heat from the GR could be accumulated in N3. After that, it was confirmed that the accumulated heat was also kept in the N3, thus, the temperature difference of each layer decreases as the temperature rises. In addition, since the nonwoven fabric that has better heat diffusion and heat transfer capability than the woven fabric structure was used as the outer layer fabric, it was represented that the surface temperature easily increased, and the temperature difference decreased, compared with that of the other outer layer fabrics. Therefore, the temperature difference between GR and each layer of the graphene-based multi-layer fabric heating element was different, according to the type of outer fabric. Also, when the fabric using AR_NW as the outer layer fabric was used, it was confirmed that the thermal insulation properties were excellent, due to the smallest difference of temperature.

In addition, when compared by relative humidity conditions, it was found that the ΔT value of the graphene-based multi-layer fabric heating element tended to decrease more under 55% RH than under 25% RH condition. The ΔT values of the graphene-based multi-layer fabric heating element with LD_ARW indicated similar value under (25 and 55) % RH condition. In the case of HD_ARW used as the outer for samples, the temperature difference between GR and N3 was increased by about 7.0 °C, whereas those of the lining and outer were similar. In addition, when the AR_KT was used as outer fabric for the graphene-based multi-layer fabric heating element, the values of ΔT_(GR-Lining)_ and ΔT_(GR-N3)_ were similar to the sample with 25% RH, but ΔT_(GR-Outer)_ decreased by about 3.0 °C. In the case of AR_NW used as the outer of samples, it was confirmed that the value of ΔT_(GR-N3)_ showed similar, but ΔT_(GR-Lining)_ and ΔT_(GR-Outer)_ decreased by about (5.0 and 10.0) °C, respectively.

The results of the present study showed that the tendency of temperature difference of each layer of the four samples at 55% RH was lower than that at 25% RH. In particular, the ΔT_(GR-Outer)_ of the four graphene-based multi-layer samples at 25% RH was about 33.0 °C or more. It was confirmed that the heat loss to the outside increases as the moisture content of the air decreases. It is generally known that moisture, that is, relative humidity, which is one of the external environmental factors, varies in thermal conductivity between clothing and human skin, and heat loss between clothing and the outside at the same temperature by the moisture content in the air^26^. It was found that the lining and fillers, which are in direct contact with GR, were similar, but the temperature difference was reduced, due to the decrease of the surface temperature with rapid heat loss in the outer layer. In addition, as described above, it was confirmed that the temperature difference between the GR and each layer of fabrics varied, depending on the thickness, density, and structure of the fabric in the multi-layer design. Based on the above results, a paired *t*-test was conducted to confirm the difference of the four samples according to the difference outer types of graphene-based multi-layer fabric heating elements at 25% RH and 55% RH in this study. The results are shown in Table 1. As a result of the analysis, when four types of aramid-based fabrics LD_ARW, HD_ARW, AR_KT and AR_NW were used as outers, the samples showed a significant difference as *p*-value was 0.00 under the environmental conditions of 25% RH and 55% RH (P < 0.05). When LD_ARW and HD_ARW were used as outer fabrics, the temperature deviation between GR and lining was low at 55% RH, and GR-filler and GR-outer were high at 55% RH. On the other hand, when AR_NT or AR_NW was used as the outer fabric, the temperature deviations of GR-lining, GR-filler and GR-outer were all indicated the higher value at 55% RH. This tendency showed the same trend as the above-mentioned result. Therefore, when the aramid nonwoven with fine fiber was applied as an outer layer fabric of sample under the environmental conditions of 55% RH, it was confirmed that the temperature difference with GR of each layer was reduced to show excellent thermal insulation performance.

**Table 1:** Results of the paired t-test analyses of graphene-based multi-layer fabric heating elements under four types of difference at 25% RH and 55% RH.

Types of outer	ΔT of layers	R.H. (%)	Mean	S.D.	*p*-value
LD_ARW	GR-Lining	25	25.17	0.09	0.00
		55	24.52	0.11	
	GR-Filler	25	15.52	0.07	0.00
		55	17.44	0.07	
	GR-Outer	25	33.54	0.08	0.00
		55	33.66	0.12	
HD_ARW	GR-Lining	25	25.37	0.09	0.00
		55	24.91	0.11	
	GR-Filler	25	15.10	0.18	0.00
		55	21.08	0.08	
	GR-Outer	25	35.99	0.43	0.00
		55	36.47	0.24	
AR_KT	GR-Lining	25	25.37	0.09	0.00
		55	24.51	0.11	
	GR-Filler	25	17.49	0.24	0.00
		55	15.10	0.18	
	GR-Outer	25	35.99	0.43	0.00
		55	33.90	0.12	
AR_NW	GR-Lining	25	22.17	0.09	0.00
		55	15.51	0.11	
	GR-Filler	25	14.81	0.07	0.00
		55	13.27	0.07	
	GR-Outer	25	26.98	0.06	0.00
		55	26.96	0.17	

## Conclusion

The object of this paper was to make a basic study of applying graphene coated fabric heating element to clothing worn in extreme and cold environment. The graphene-based multi-layer fabric heating elements were designed as a layer of outer, filler, electrical heating textile, and lining. After that, the thermal insulation properties were measured based on the electrical heating test by various layer numbers and layer types. Each sample was composed of double-layer, triple-layer, and quadruple-layer based on graphene-based electrical heating textile (below GR) as single-layer, and then the temperature change and difference of each layer was examined.

First, in this study, GR, which has a surface temperature of about 85 °C, a current of 0.12 A, and a power of 3 W when 30 V is applied, was prepared. As a result of analyzing the surface temperature after layering with each designed multi-layer, it was confirmed that the temperature of the double-layer of lining/GR increased about 5 °C. In the case of triple-layer based on lining/GR, by adding lining, the surface temperature was found to be over 60 °C, and it was increased by about 5 °C compared with double-layer with non-lining, due to the increased amount of air content by the naps in the lining, which can entrap the warm air in the layer. In the case of quadruple-layer based on lining/GR/N3, the surface temperature was about 50 °C, and different surface temperature was confirmed, depending on the fabric used as the outer. When the aramid woven fabrics were used as the fabric having excellent heat resistance, the surface temperature was low at about 45 °C. On the other hand, when the aramid nonwoven was used, it was also made by aramid fiber, but it had fine fiber and formed high porosity, so that the warm air could be entrapped within the high porosity, thus the temperature was the highest at about 51 °C. As a result of the temperature of each layer of 6-types of graphene-based multi-layer fabric heating elements, the temperature was higher in the order of N3 > Lining > Outer located close to the GR layer. According to the outer layer fabric, the inside and outside temperature of the outer layer appeared different; and when AR_NW was used, the temperature of Lining, N3, and the outer was higher, than when the other fabrics were used as outer layer fabric. The thermal insulation performance was examined by measuring the temperature of each layer on the basis of the electrical heating test, and calculating the temperature difference from the GR. As a result, the temperature difference of each layer was in the order ΔT_(GR-N3)_ < ΔT_(GR-Lining)_ < ΔT_(GR-Outer)_; it tended to decrease in the order of close to the layer of GR. It was confirmed that this was because of the easy heat transfer and low heat loss from GR, which produced direct heat. In particular, when AR_NW was used as the outer layer fabric, the temperature difference decreased by about 10 °C, compared with that of the other outer layer fabric, confirming that the fabric structure affected the multi-layer of samples. In addition, it was confirmed that the thermal insulation performance was increased under 55% RH condition, which is more comfortable than the dry state. Therefore, this present study developed a quadruple-layer graphene-based multi-layer fabric heating element through various fabric designs based on graphene-based electrical heating textile and analyzed the thermal insulation performance based on electrical heating test. We expect to expand its application to smart electrical heating textiles and wearable electrical heating devices that can actively maintain body temperature in outdoor, military, or protective clothing.

## Methods

In this study, graphene (GNP-UC, Carbon nano technology Co. Ltd., Korea) and poly(vinylidene fluoride-co-hexafluoropropylene) chips (PVDF-HFP, SOLEF 21508, Solvay Co. Ltd., Belgium) were the same as those used in the previous study. The 1st grade acetone (Junsei Chemical Co. Ltd., Japan) was used as solvent. Flame retardant (below FR) cotton fabric that consisted of 0.65 mm thickness, 0.042 g/cm^2^ density, and twill structure, was used as substrate material. Samples were sewn by industrial sewing machine (LS2-B736, Unicorn, Korea) with both silver-coated conductive yarn (140 D, Soitex, Korea) at the upper used as electrode, and commercial cotton yarn of 280 D at lower. Table 2 indicates the sample code and specifications of the used fabrics. The various types of textile materials were used to create the graphene-based multi-layer fabric heating element. Four kinds of aramid materials, neoprene fabric, and waterproof brushed fabric were collected and used. For the outer materials, the aramid materials were two types of different densities aramid woven fabrics, an aramid knit and aramid nonwoven fabric.

**Table 2:** Sample code and specifications of the used fabrics.

Sample code	Type	Thickness (mm)	Weight (g/cm^2^)	Images		Used part
				Surface	Back side	
LD_ARW	Aramid woven	0.22	0.019			Outer
HD_ARW		0.28	0.013			
AR_KT	Aramid knit	1.00	0.018			
AR_NW	Aramid nonwoven	1.00	0.012			
N3	Neoprene	3.00	0.061			Filler
WPB_F	Waterproof Brushed fabric	1.00	0.033			Lining

First, to fabricate the graphene/PVDF-HFP composite coated on fabric with horseshoe pattern, 16 wt.% graphene/PVDF-HFP composite solution was obtained by our previous study^24^. The PVDF-HFP chips were dissolved in 1st grade acetone to prepare a 15 wt.% PVDF-HFP solution, and then 16 wt.% graphene was added to the fabricated PVDF-HFP solution. The 16 wt.% graphene/PVDF-HFP composite solution was stirred for a week with a digital controlled hotplate stirrer (MSH-20D, Daihan Scientific, Korea). The graphene/PVDF-HFP coated on flame-retardant (below FR) cotton fabric with horseshoe pattern (below HP) type was also prepared, as in our previous study^24^. To prepare the electrical heating textile with HP, the HP was designed with a width of 50 mm and a length of 2.5 mm, respectively. Six lines of HP were arranged in an area of 50 mm × 50 mm. Then, untreated FR cotton fabric was prepared in the size of 60 mm × 60 mm. Silver-coated conductive yarn, which was used as electrodes, was sewn in three straight lines within 5 mm on both sides of the untreated FR cotton fabric. After that, 6 lines of HP were coated on the FR cotton fabric using the knife edge method with 16 wt.% graphene/PVDF-HFP composite, and then samples were hot-pressed at 140 °C for 3 min at 3.5 MPa. The surface temperature, current, and power by DC power supply (Keithley 2260B-250-4, Tektronix, Inc., USA) of the as-fabricated samples were measured. Finally, a sample showing a surface temperature of about 85 °C, a current of 0.12 A, and a power of 3 W when 30 V was applied was obtained. Samples were stored in a desiccator before characterization.

To fabricate the graphene-based multi-layer fabric heating element, different textile materials have been applied to graphene/PVDF-HFP coated electrical heating textile (below GR). In general, a multi-layer heating textile element, which is used for protective clothing protecting against cold, is designed as a lining/filler/outer shell fabric. In this study, the graphene-based multi-layer fabric heating element according to various fabric layers was designed from single-layer to quadruple-layer, as follows: (1) single layer: GR, (2) double-layer: Lining/GR, GR/Outer, or GR/Filler (3) triple-layer: Lining/GR/Outer or Lining/GR/Filler, (4) quadruple-layer: Lining/GR/Filler/Outer. Table 3 indicates the scheme of measuring layer of graphene-based heating element with various fabric layers.

**Table 3:** Measuring layer of various types of graphene-based multi-layer fabric heating elements.

Number of layers	Materials	Images	Type of outer	Thickness (mm)
1 single	GR		–	0.78 ± 0.01
2 double	Lining/GR		–	1.78 ± 0.01
	GR/Filler		–	3.78 ± 0.01
	GR/Outer		LD_ARW	1.00 ± 0.01
			HD_ARW	1.06 ± 0.01
			AR_KT	1.78 ± 0.01
			AR_NW	1.78 ± 0.01
3 triple	Lining/GR/Filler		–	4.78 ± 0.01
	Lining/GR/Filler		LD_ARW	2.00 ± 0.01
			HD_ARW	2.06 ± 0.01
			AR_KT	2.78 ± 0.01
			AR_NW	2.78 ± 0.01
4 quadruple	Lining/GR/Filler/Outer		LD_ARW	5.00 ± 0.01
			HD_ARW	5.06 ± 0.01
			AR_KT	5.78 ± 0.01
			AR_NW	5.78 ± 0.01
			

Finally, to confirm the effect of relative humidity on the graphene-based multi-layer fabric heating element, samples with quadruple-layer of aramid fabric, which was used as outer, were prepared. The relative humidity conditions followed the KS K 0589 standard. According to this standard, a desiccator was controlled with water-sulfuric acid solution having 25% RH, which is dry condition, or 55% RH, which is comfortable condition, at 20 ℃. Before treatment with each relative humidity condition, samples were dried at 105 ℃ in the dry oven, and then stored in the controlled desiccator, until test. This condition was set as initial state in this study.

**Figure 5:**
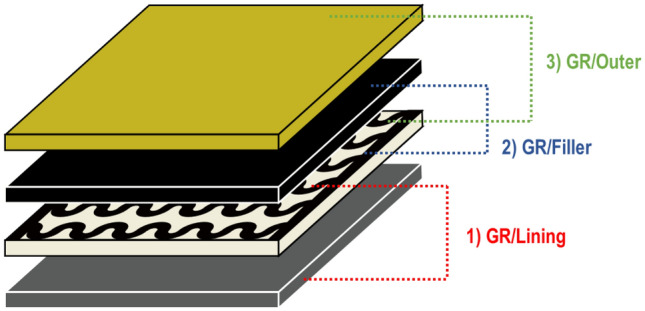
Measured layer for thermal insulation property based on electrical heating test.

To investigate the electrical heating performance according to the number of layers and fabric types, the surface temperature of the upper layer when the voltage was applied to the GR after placing each layer was measured. Electrical heating performance was measured with a DC power supply (CPS-2450B, Chungpa EMT Co. Ltd., Korea). The ( +) and (−) poles of the DC power were connected with alligator clips. These alligator clips were linked in the diagonal direction of two ends of the GR sample that was sewn as silver-coated conductive yarn. The applied voltage range was from (0 to 30) V at 5 V (DC) interval for 3 min. Temperatures of the upper surface of samples were measured by thermal imaging camera (FLIR i5, FLIR Systems INC, USA). Current values were measured with a DC power supply when voltage was applied to the sample. Mean value was obtained from three measurements per sample. To confirm the temperature and electrical heating behavior of the layer inside the graphene-based multi-layer fabric heating element by the number of layers and types of fabric, the electrical heating behavior was measured with a temperature data logger (TR-71wf, T&D Corp., Japan), and a temperature sensor (TR-0206, T&D Corp., Japan). First, a graphene-based multi-layer fabric heating element was designed with four layers according to the type of outer fabric, and a temperature sensor was placed between each layer. After that, the alligator clips connected to the DC power supply were connected to the two edges of the GR sample, and an applied voltage was applied. At this time, 30 V, which is a condition that the surface temperature of the quadruple-layer indicated 50 °C, was applied. To confirm the electrical heating behavior of samples, 30 V was applied to each sample for 30 min. Temperature behavior was measured until the temperature reached equilibrium state. In addition, the thermal insulation properties were measured based on the electrical heating test in this study. The difference of temperature between the GR layer that shows the direct heating performance in graphene-based multi-layer fabric heating elements and the lining, filler, and outer was calculated. The three multi-layer values, ΔT_(GR-Lining)_, ΔT_(GR-Filler)_, and ΔT_(GR-Outer)_, were measured, as shown in Fig. 5. The analysis of variance test for the test results was carried out by SPSS (statistical package for social science) Win version 26.0 to analyze the temperature difference of four types of graphene-based multi-layer fabric heating elements with various outer fabrics at 25% RH and 55% RH condition. A paired t-test was used to analyze the correlation between different outer fabrics and different relative humidity test conditions. p < 0.05 was considered significant.
